# Stage at diagnosis and tumor characteristics among young women and men with breast cancer, in Ethiopia and Sweden, a descriptive cross-sectional study

**DOI:** 10.1186/s12885-025-14614-x

**Published:** 2025-07-27

**Authors:** Tove Ekdahl Hjelm, Tewodros Yalew Gebremariam, Mahlet Fekadu Weldearegay, Moti Sori, Marcus Bauer, Bethlehem Ayele Getachew, Mathewos Assefa, Endale Anberber, Hidaya Yahya Mohammed, Eva Johanna Kantelhardt, Sara Margolin, Annika Lindblom, Senait Ashenafi, Jenny Löfgren

**Affiliations:** 1 Department of Molecular Medicine and Surgery, Karolinska Institute, Stockholm, Sweden; 2 Department of Oncology, Södersjukhuset, Stockholm, Sweden; 3 Department of Pathology, School of Medicine, College of Health Sciences, Tikur Anbessa Specialized Hospital, Addis Ababa University, Addis Ababa, Ethiopia; 4 Institute of Pathology, Martin Luther University, Halle-Wittenberg, Halle, Germany; 5 Department of Oncology, School of Medicine, College of Health Sciences, Tikur Anbessa Specialized Hospital, Addis Ababa University, Addis Ababa, Ethiopia; 6 Department of Surgery, School of Medicine, College of Health Sciences, Tikur Anbessa Specialized Hospital, Addis Ababa University, Addis Ababa, Ethiopia; 7 Global Health Working Group, Institute of Medical Epidemiology, Biometrics, and Informatics, Martin-Luther-University Halle-Wittenberg, Halle, Germany; 8 Department of Gynaecology, Martin-Luther-University Halle-Wittenberg, Halle, Germany; 9 Department of Clinical Science and Education Södersjukhuset, Karolinska Institute, Stockholm, Sweden; 10 Department of Clinical Genetics and Genomics, Karolinska University Hospital, Stockholm, Sweden; 11 Department of Reconstructive Plastic Surgery, Karolinska University Hospital, Stockholm, Sweden

**Keywords:** Early-onset breast cancer, Male breast cancer, Sub-Saharan Africa, East Africa, Tumor stage, Tumor subtype

## Abstract

**Background:**

Breast cancer patients diagnosed in sub-Saharan Africa (SSA) are generally younger, and present with more advanced stage of disease, than those in high-income countries. In addition, male breast cancer appears to be more prevalent in SSA. Young women and men are typically not included in national mammography screening programs. Therefore, the aim of the present study was to compare clinical and pathological data from a breast-cancer-patient cohort not covered by mammography screening, in a low-income country in SSA (Ethiopia), to a similar patient cohort from a high-income country in Europe (Sweden).

**Methods:**

Women (< 40 years) and men (all ages) with breast cancer were recruited in Ethiopia and Sweden. Patient- and tumor data was collected. In Ethiopia, 100 study participants were recruited prospectively from the Departments of Surgery and Oncology at Tikur Anbessa Specialized Hospital. In Sweden, 100 study participants were enrolled retrospectively from the Department of Oncology at Södersjukhuset, Stockholm.

**Results:**

Ethiopian and Swedish study participants were diagnosed in tumor stage I (3.3% vs 27.0%), stage II (33.7% vs 45.0%), stage III (44.6% vs 23%), and stage IV (18.5% vs 5.0%). This represents a significant difference in stage distribution between groups (*p* < 0.001). A majority of the cases were ER-positive (79.5% in Ethiopia and 69.0% in Sweden, *p* = 0.08). The ER- and/or PgR-positive/HER2-negative subtype was the most common in both groups: (68.0% in Ethiopian patients and 47.5% in Swedish patients). The HER2-positive (any ER) subtype accounted for 20.5% in Ethiopia and 26.7% in Sweden, while triple-negative breast cancer accounted for 11.5% (Ethiopia) and 25.7% (Sweden).

**Conclusions:**

There were large disparities in stage at diagnosis between Ethiopian and Swedish young women and men with breast cancer, with a higher proportion of late-stage disease seen in Ethiopians although, due to young age/male sex, none of the Swedish cases were diagnosed in the national mammography screening program. There was a high rate of ER-positive breast cancer at both sites, and the triple-negative subtype was more than twice as common in Swedish patients.

## Background

Breast cancer is the most common type of cancer in women worldwide, with an estimated 2.3 million new cases and 685,000 deaths per year [1]. Even though incidence rates are higher in high-income countries (HIC) than in low-middle-income countries (LMIC), mortality rates in LMICs are much higher, reflecting a lower survival outcome and global inequities in cancer care [1, 2].

In general, young women with breast cancer have worse survival outcomes and a greater risk of recurrence, than do older women [3]. The prognosis is highly dependent on the stage at diagnosis as well as on the tumor grade and subtype of cancer. Young women usually present with larger tumor size and aggressive tumor biology, with a higher proportion of luminal B, human epidermal growth factor receptor 2 (HER2)-positive, or triple-negative breast cancer (TNBC) [4]. Notably, even in early-stage disease, the risk of dying of breast cancer is more than tripled among women under the age of 40 compared to women above 40 years [3, 5].

The risk of developing breast cancer is multi-factorial, female sex and higher age being the most important risk factors. Inherited-, hormonal-, environmental-, life-style- and breast-related factors all contribute to the individual risk. For early-onset breast cancer, the most important risk factor is genetic predisposition [4, 6]. Breast cancer patients in Sub-Saharan Africa (SSA) are in general younger when diagnosed than those in HIC, likely due to the young population structure, and they often present with more advanced stages of disease [7–9]. A meta-analysis of 83 studies with 24,213 breast cancer patients in SSA demonstrated that 77% were diagnosed at tumor stages III-IV [10]. Breast cancer mortality rate by the age of 40 varies widely between continents, and in Africa, the mortality rate by the age of 40 is more than double that of the global average [11].

Ethiopia is a low-income country (LIC) in East Africa. It is a populous country of over 120 million inhabitants (2021) [12]. As in many LICs in SSA, breast cancer patients in Ethiopia are diagnosed at a young age with a mean age of 43 years. A majority of patients in the age span of 30–39 years old has been described [13]. Male breast cancer patients represent a larger proportion of diagnosed cases, with around 6% [14–16], compared to less than 1% of all breast cancer cases in HIC [17].

In the present study, early-onset breast cancer in women, and breast cancer in men were investigated in a LIC setting in East Africa (Ethiopia) and a HIC setting in northern Europe (Sweden). Ethiopia was chosen as LIC study site due to the known high proportion of young women and men diagnosed with breast cancer, and in addition, collaborating partnership had been developed with the Pathology Department at the Tikur Anbessa Specialized Hospital (TASH), Addis Ababa and Karolinska Institutet, Sweden, making the study feasible. The aim was to compare potential differences and similarities of tumor stage at diagnosis, tumor characteristics, and presence of risk factors, in a breast-cancer-patient cohort not covered by mammography screening programs.

### Significance

Increased understanding of the presentation of breast cancer in Ethiopia, and potential differences compared to the Swedish cohort, could help to guide further studies and interventions for early detection. Insight into the distribution of breast cancer subtypes in the Ethiopian setting, has the potential to reduce mortality if personalized treatment would be made available for more patients, and could potentially be used to advocate for increased availability of targeted therapy.

## Methods

### Study design

This was an observational, descriptive cross-sectional study with prospective data collection in Ethiopia and retrospective, electronic file-based data collection in Sweden.

### Selection and description of study sites and participants

Study participants were recruited from two sites, Ethiopia, a LIC in East Africa, and Sweden, a HIC in northern Europe. From each site, 100 study participants were included.

The Ethiopian study participants were recruited from TASH in Addis Ababa. This is the National Referral Hospital, the largest hospital in the country and the only one with available radiotherapy units when the present study was initiated. Basic breast cancer care including surgery, basic chemotherapy, radiotherapy and hormonal treatment such as Tamoxifen, is available, although, lack of resources can lead to long waiting time for treatment initiation, and shortages of medicines and equipment are common [18]. Additional investigations and treatments such as core needle biopsy for pathological diagnosis and immunohistochemistry (IHC) were not routine in the public health care facilities when the participants were included in the study. These additions were provided by the research project to the study participants free of charge.

Inclusion criteria were women 18–39 years, and men over 18 years, with confirmed invasive breast cancer, residing in or around Addis Ababa (within a 100 km catchment area), and who were willing to participate. Exclusion criteria were unverified cancer and unwillingness or inability to give informed consent.

The Swedish study participants were recruited from Södersjukhuset (SÖS), a public hospital in Stockholm. The hospital has the largest breast oncology unit in Sweden with over 800 new breast cancer cases per year [19]. Comprehensive breast cancer care is delivered within the publicly funded healthcare system. Women between 18 and 39 years, and men 18 years and above who had been diagnosed with invasive breast cancer and managed according to local guidelines, were included. In Sweden, there are rigid protocols for tissue handling, a high quality of samples, as well as available electronic charts with direct access to all patient data, including radiology- and pathology reports. Hence, the retrospective approach in this setting was considered sufficient for comparison.

### Data collection and measurements

At TASH, Ethiopia, study participants were recruited prospectively from February 2021 until the sample size was reached in September 2023. Patients eligible for inclusion and who presented at the Departments of Oncology or Surgery at TASH were offered participation. Data was collected using a) a questionnaire for patient characteristics, medical history and presence of known risk factors for breast cancer, b) a biopsy from the breast lesion and/or surgical specimen used for pathologic evaluation for diagnostics including immunohistochemistry analysis, and c) clinical (c)TNM stage at diagnosis and radiology reports obtained from patient charts. Potential study participants were offered a session of breast health education with individual, face-to-face information and written information. This was provided by a trained physician in English or Amharic, depending on the patient’s preference. Before inclusion, patients were asked to sign or thumbprint an informed consent form. For a majority of the study participants, tissue samples were collected directly from the operation theater by members of the research team to ascertain correct handling including timely transport to the pathology department and fixation in formalin.

Pathological assessment and IHC were performed locally in the Department of Pathology at TASH. IHC methods were established in collaboration with a third partner at the Institute of Pathology, Martin Luther University, Halle-Wittenberg, Germany, that validated the IHC results by regular online- and on site meetings. Either resection tissue samples or core-needle biopsies were used. Tissue samples were fixed with 10% neutral buffered formalin solution. The formalin-fixed, paraffin-embedded blocks were cut to 4–5 µm when used for hematoxylin and eosin (H&E) staining, and to 3 µm for IHC tissue staining. Samples were analyzed by histomorphology using H&E staining for histological diagnosis according to the World Health Organization (WHO) classification of breast tumors, 5th Edition, 2019. Nottingham grading (NHG) was determined according to Elston and Ellis [20]. All samples were analyzed by conventional IHC using a manual staining procedure (ZytoChem Plus HRP Polymer System, Zytomed Systems, Germany). For all cases, antibodies directed against the estrogen receptor (ER), progesterone receptor (PgR) and HER2 were employed. Expression of ER and PgR status was analyzed according to current guidelines [21]. A negative ER or PgR status was declared as receptor expression of < 1% of tumor cells. If at least one of the markers was positive, the hormone receptor status was defined as positive. HER2 status was assessed according to the American Society of Clinical Oncology and College of American Pathologists (ASC-CAP) guidelines [22].

At SÖS, Sweden, retrospective data collection was performed during September 1, 2023, until December 1, 2023. Eligible patients diagnosed between January 1, 2020, until October 5, 2022, were included consecutively until 100 study patients were reached. Electronic charts were reviewed for basic patient characteristics, medical history, presence of known risk factors for breast cancer, TNM stage at diagnosis, tumor biology including pathology, and radiology reports. Data was extracted and entered in Excel spreadsheets before analysis.

### Definitions

ER and PgR were defined as positive when ER/PgR were ≥ 1%, in both Ethiopia and Sweden. (However, in the national Swedish guidelines ER and PgR are required to be ≥ 10% to be positive [23]). These cut-off levels were aligned to the criteria in the TASH Pathology Department in order to make comparison possible. In Sweden HER2-positive was defined as either HER2 3 + on IHC or HER2 2 + on IHC, verified as positive using silver-enhanced in situ hybridization (SISH). In Ethiopia, in situ hybridization (ISH) for verification of HER2 2 + cases were not available, and only HER2 3 + cases were defined as HER2-positive.

Tumors were staged according to the eight edition of the American Joint Committee on Cancer where the anatomical tumor node metastasis (TNM) system was used, biomarkers and Oncotype DX were not used [24]. When available, pathological (p)TNM was used; if unavailable, cTNM was used instead. Since the sample size was small, in the final analysis study patients were grouped into stages I-IV. IHC receptor status was used as a surrogate marker for biological subtypes and the study patients were grouped in the analysis as luminal-like (ER- and/or PgR-positive, HER2-negative), HER2-positive (HER2-positive and ER-positive or ER-negative) and TNBC (ER-negative, PgR-negative, and HER2-negative).

### Statistics

Pearson’s chi-squared test and Fisher´s exact test were used to test for a statistically significant difference between the observed frequencies in the studied variables in the groups. A significance level of 5% (*p* < 0.05) was used.

## Results

In Ethiopia, 101 study patients were enrolled, out of which one patient later withdraw consent and was excluded leaving 100 patients for analysis. One hundred patients were recruited from Sweden. The mean age of the female patients was 33.3 years in Ethiopia and 34.8 years in Sweden. Six males were included in Ethiopia and eight in Sweden, with a mean age of 49 years (Ethiopia) and 64.5 years (Sweden), see Table 1.

**Table 1:** Basic characteristics of study participants in Sweden and Ethiopia

Clinical Characteristics	Swedish participants (*n* = 100)	Ethiopian participants (*n* = 100)	*p*-value
Age			
Female patients, mean/median (range) Range	34.8/35 (23–39)	33.3/35 (21–39)	
< 25	2	2	*p* = 0.070
25–29	4	15	
30–34	33	27	
35–39	53	50	
Male patients, mean/median (range)	64.7/64.5 (21–83)	58.3/49 (40–76)	
Gender			
Female	92	94	*p* = 0.579
Male	8	6	
Pregnancy carried to term (female patients)			
Yes, n	67	61*	
1–2 children	54	42*	*p* = 0.271
3 or more children	13	19	
No, n	25	27	
Unknown	0	6	
Breast feeding, n (%) (females with children)			
Yes	45 (97.8)	55 (90.2)	*p* = 0.235
No	1 (2.2)	6 (9.8)	
Unknown	21	0	
Use of oral contraceptives (ongoing or previous), n (%)			
Yes	34 (65.4)	14 (15.7)	*p* < 0.001
No	18 (34.6)	75 (84.3)	
Unknown	40	5	
Smoking, n (%)			
Yes	11 (11.5)	1 (1)	*p* = 0.002
No	85 (88.5)	99 (99)	
Unknown	4	0	
Drinks alcohol (any), n (%)			*p* < 0.001
Yes	58 (67.4)	5 (5.1)	
No	28 (32.6)	94 (94.9)	
Unknown	14	1	
Way of cancer detection			
Self-detection	94	93	-
Mammogram**	2	0	
Clinically detected	2	0	
Referral from emergency unit	2	0	
Referral dermatologist	1	0	
Unknown	0	7	
Symptoms leading to seeking health care***			
Lump	87	83	-
Wound	1	7	
Nipple discharge	4	4	
Discomfort	7	7	

^*^One patient had one stillbirth

^**^One patient did a control mammogram due to earlier DCIS; another did a control mammogram for known fibroadenoma

^***^Patients could have more than one symptom leading them to seek health care

Almost all cancers were self-detected by the study participants: in Ethiopia 100% (of known) vs 94% in Sweden. Finding a lump in the breast was the most common symptom leading to seeking health care in both study settings, followed by discomfort. Ductal cancer was the most common histological type in both countries (96.0% in Ethiopia and 91.8% in Sweden). Histological grade (NHG) was missing for 28 of the Ethiopian patients. For those with available results, the majority, 45.8% (*n* = 33), were NHG II cancers. In Sweden, the majority, 62.6% (*n* = 57), were NHG III (Table 1).

Ethiopian patients were diagnosed with larger tumors, with 7.9% (*n* = 7) T1 tumors and 27% (*n* = 24) T4 tumors, compared to 38.4% (*n* = 38) T1 tumors and 4.3% (*n* = 4) T4 tumors in Sweden. In both settings, there was a high proportion of node-positive disease at diagnosis, with a proportion of lymph-node-positive disease of 75.0% in Ethiopia and 50.5% in Sweden. In Ethiopia, 18.5% (*n* = 17) had metastatic disease at diagnosis, compared to 5.0% (*n* = 5) in Sweden. Six Ethiopian patients included were shown to have recurrent disease (Table 2).

**Table 2:** Tumor characteristics and stage at diagnosis

Tumor Characteristics	Sweden, *n* = 100 n (%)	Ethiopia, *n* = 100 n (%)	*p*-value
Histology type			-
Invasive breast carcinoma—NST (no special type	89 (91.8)	96 (96.0)	
Lobular carcinoma	0 (0)	2 (2.0)	
Medullary carcinoma	0 (0)	0 (0)	
Mucinous carcinoma	3 (3.1)	1 (1.0)	
Mixed carcinoma	3 (3.1)	0 (0)	
Other (secretory/pleomorphic/papillary)	2 (2.1)	1 (1.0)	
Unknown/not specified	3	0	
Tumor Size* all (%) female/male	´		*p* < 0.001
T1 (< 20 mm)	38 (38.4) 35/3	7 (7.9) 7/0	
T2 (> 20–50 mm)	44 (44.4) 40/4	33 (37.1) 30/3	
T3 (> 50 mm)	13 (13.8) 13/0	25 (28.1) 23/2	
T4 (extension to chest wall, skin, ulceration, inflammatory cancer)	4 (4.3) 3/1	24 (27.0) 24/0	
Unknown/missing	1	5	
Recurrent disease	0	6	
Lymph node status* all (%) female/male			*p* < 0.001
N0	49 (49.5) 44/5	21 (24.7) 19/2	
N1	36 (36.4) 34/2	25 (29.4) 22/3	
N2	9 (9.1) 8/1	24 (29.4) 24/0	
N3	5 (5.1) 5/0	14 (16.5) 14/0	
Unknown/missing	1	10	
Recurrent disease	0	6	
Distant metastasis* all (%) female/male			*p* = 0.01
M0	66 (66.0) 63/3	62 (65.9) 58/4	
M1	5 (5.0) 4/1	17 (18.1)16/1	
Mx	29 (29.0) 25/4	15 (16.0) 14/1	
Recurrent disease	0	6	
Histological grade (NHG) all (%)			-
I	3 (3.3)	11 (15.3)	
II	31 (34.1)	33 (45.8)	
III	57 (62.6)	28 (38.9)	
Unknown/missing	9	28	
Estrogen receptor status (ER > 1%) all (%) female/male			*p* = 0.080
Positive	69**(69) 62/7	62 (79.5) 58/4	
Negative	31 (31)/30/1	16 (20.5) 16/0	
Unknown/missing	0	22 20/0	
Tumor subtype (%)			*p* = 0.014
Luminal-like	48 (47.5)	53 (68.0)***	
HER2-positive, luminal/non-luminal-like	27 (26.7)** 22/5	16 (20.5) *** 11/5	
Triple-negative breast cancer	26 (25.7)	9 (11.5)	
Unknown/missing	0	22	
Tumor stage (UICC) at diagnosis all (%) female/male			*p* < 0.001
I	27 (27.0) 26/1	3 (3.3) 3/0	
II	45 (45.0) 41/4	31 (33.7) 28/3	
III	23 (23.0) 21/2	41 (44.6) 39/2	
IV	5 (5.0) 4/1	17 (18.5) 16/1	
Unknown/missing information	0	2	
Recurrent disease	0	6****	

^***^ For the majority pTNM was used, when not available cTNM was used instead

^**^One patient had bilateral breast cancer with one cancer of the luminal subtype and contralateral cancer of the HER2-positive subtype

^***^Four patients were HER2 2 +, and two patients had ER-/HER2-negative and PgR-positive biology

^****^Six patients included had previous breast cancer and presented with recurrent disease

**Fig. 1:**
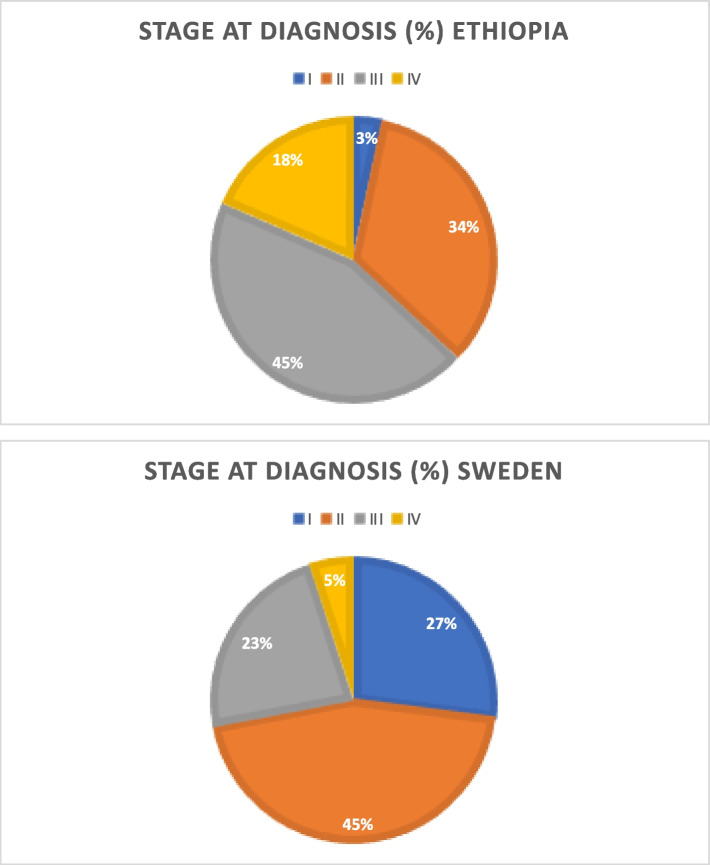
Stage at breast cancer diagnosis in Ethiopia and Sweden (all patients)

There was a statistically significant difference in stage distribution at diagnosis between the two countries. Ethiopian and Swedish study participants were diagnosed at tumor stage I 3.3% vs 27.0%, stage II 33.7% vs 45.0%, stage III 44.6% vs 23%, and stage IV 18.5% vs 5.0%, respectively (*p* < 0.001). In Ethiopia, the majority (78.3%) presented in stages II and III, while in Sweden the majority (72.0%) were diagnosed at stages I and II, see Table 2, Fig. 1.

**Fig. 2:**
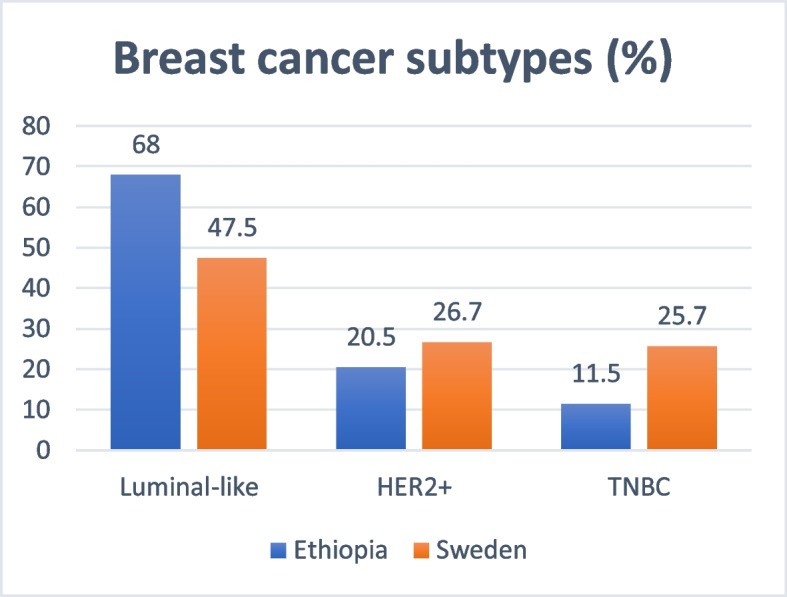
Breast cancer subtypes by immunohistochemistry, Ethiopia and Sweden (all patients); Luminal-like: ER-positive and HER2-negative

In both countries, the majority of cancers were ER-positive, 79.5% in Ethiopia and 69.0% in Sweden. There was no statistically significant difference between the groups regarding ER-status (*p* = 0.080). The luminal-like subtype was the most common in both countries with 68.0% in Ethiopia and 47.5% in Sweden, followed by HER2-positive (luminal or non-luminal), 20.5% in Ethiopia and 26.7% in Sweden. In Ethiopia, the proportion of TNBC was less than half that of the Swedish patients (11.5% and 25.7% respectively), there was a statistically significant difference in subtype distribution between groups (*p* = 0.014), Table 2, Fig. 2).

Some modifiable risk factors for breast cancer varied between groups, with alcohol intake (67.4% in Sweden vs 5.1% in Ethiopia, *p* < 0.001), use of hormonal contraceptives (65.4% in Sweden vs 15.7% in Ethiopia, *p* < 0.001), and smoking (11.5% in Sweden vs 1.0% in Ethiopia, *p* = 0.002), being more prevalent in Swedish patients.

## Discussion

This study demonstrates differences in stage at diagnosis between the study sites, with Ethiopian patients presenting with more advanced cancer than Swedish patients. Even though the majority of breast cancers in young women and men in both countries are self-detected, with a lump being the most common reason for seeking health care, there are disparities in stage at diagnosis. The fact that the majority of Ethiopian patients were diagnosed in the late-stage of disease, is consistent with several previous studies from SSA. For instance, population-based studies from cancer registers including 12 SSA nations found that 64.9% of patients were diagnosed with stages III-IV disease while 18.4% had metastatic disease at diagnosis [2].

Since men and women under the age of 40 years are not included in general mammography screening neither in Sweden nor in Ethiopia, other factors than screening effect must account for the difference in stage at diagnosis. More aggressive tumor subtypes in the Ethiopian setting compared to the Swedish setting does not seem to be the explanation, as luminal-like breast cancer was the most prevalent subtype in both regions. In addition, a smaller proportion of TNBC was seen among the Ethiopian patients than among the Swedish. The reasons for Ethiopian patients presenting later and with more advanced disease than Swedish patients is most likely multifactorial, and warrants further investigation to identify barriers and causes for delay. Potential contributing factors could be related to breast cancer awareness among the general population and health care workers, as well as access to appropriate and timely breast cancer diagnostics.

Several studies investigating tumor subtypes have shown a high proportion of TNBC in breast cancer patients in SSA, although the distribution of breast cancer subtypes varies widely between countries within SSA. For instance, TNBC appears to be less prevalent in East Africa than in West Africa [25, 26]. The variations in breast cancer subtypes are likely explained both by true regional differences based on genetic background and environment, but study biases such as small sample size, retrospective study design, poor sample quality and analytical methods for IHC in the studies could all affect study results [27–29]. However, there are additional evidence from the US, where studies show a lower frequency of TNBC disease in Eastern-African-born black women (majority born in Ethiopia) compared to US-born black and Western-African-born black women [30, 31].

The present study found an unexpectedly low proportion of TNBC cancer in the Ethiopian study group, with TNBC being less than half of what was seen in the Swedish group. It is possible that differences in sample handling, laboratory methods or IHC criteria interpretation play some role, leading to underestimation of TNBC prevalence. However, low rate of TNBC among young Ethiopian patients have previously been reported by Hadgu et al., with 17% TNBC in patients under age 40, and Jiagge et al., describes 15% of TNBC in a breast cancer cohort (mean age 43) [25, 32], further strengthen low TNBC rate among young Ethiopian patients. The findings of high frequency of ER-positive breast cancer in the Ethiopian study group is consistent with previous studies by Kantelhardt et al., and Hadgu et al., both reporting a prevalence around 65% of ER-positive breast cancer cases [25, 28]. It is possible risk factors contribute to the different rate of TNBC seen between the cohorts, as use of oral contraceptive pills, family history of cancer and mammographic density could increase the risk of TNBC [33], although, the role of risk factors in early onset TNBC besides family history are less certain. Specific risk factors for early onset breast cancer, such as genetic predisposition, might play an important role in Ethiopia, however, research studies investigating this are yet lacking.

The proportion of males in this study did not differ substantially between the Ethiopian and Swedish groups, likely explained by the selection of patients, i.e., under age 40 for females and all ages of males consecutively (Table 1). The mean age of male patients was lower in Ethiopia, at 49 years, than 64.5 in Sweden. However, the study groups of male patients are very small, and conclusions based on this report alone should be made with caution.

Mastectomy and axillary lymph node dissection are the mainstay of surgery provided in Ethiopia. Large tumor size and lymph node metastasis at diagnosis often require neoadjuvant chemotherapy for downstaging, to make breast-conserving surgery possible. However, LIC settings such as in Ethiopia often show a long waiting time for neoadjuvant chemotherapy, and in addition, timely access to adjuvant radiotherapy is very limited.

The high proportion of ER-positive tumors found in Ethiopia implies that hormonal therapy is an important tool for treatment in this setting, and reinforces the practice used in many SSA countries, where hormonal therapy is recommended to all patients if IHC is not available [34]. Although, this approach will still lead to overtreatment of a substantial number of patients. Unfortunately, no HER2-directed therapy is currently provided within the public health care system in Ethiopia, hence, it is not available for most patients. In the present study, over 20% of Ethiopian patients were HER2-positive, and possibly even more patients would have been defined as positive if HER2 2 + cases could have been verified with ISH analysis. Since HER2-positive breast cancer has a poor prognosis without targeted therapy [35, 36], there is an urgent need for implementation of HER2 diagnostic services as well as directed targeted therapy, and there are now ongoing training and capacity-building in the Pathology laboratory in TASH to implement ISH for HER2 verification. The implementation of IHC and in addition ISH methods for subtype analysis of breast cancer could drastically reduce mortality rates if personalized treatment were made available for more patients. There is a need for global health initiatives to advocate for increased access to Trastuzumab, and possibly an opportunity for clinical trials if pharmaceutical companies could be approached and involved.

Interestingly, certain risk factors differed considerably between Swedish and Ethiopian patients showing a higher rate of alcohol intake, use of oral hormonal contraceptives and smoking among the Swedish whereas fertility rates and breast-feeding did not differ much. and calls for more investigations of the contribution of risk factors for early-onset breast cancer in the Ethiopian setting.

### Strengths

The strength of the present study is its design, with prospective data collection in Ethiopia. Efforts were made to increase quality of tissue samples and analyses for tumor biology. Knowing there is a risk of tissue and protein degradation due to inadequate tissue handling in current practice in many SSA countries, retrospective results of proportions of tumor subtypes should be interpreted with caution. This study included capacity-building, by setting up IHC methods locally in the Department of Pathology at TASH, and included training abroad for pathologists, and consecutive online discussions, to increase sustainability after the study was finished. At TASH, study participants were recruited from both Departments of Surgery and Oncology, and therefore patients at stage IV disease were also included and evaluated. These patients do not routinely undergo surgery and most diagnosis is made using cytology material in the clinical setting, consequently, tumor material is often not available for evaluation.

### Limitations

The small sample size of the present study, with only one center per country, represents a limitation, potentially affecting the generalizability of the findings. TASH is the national referral center for breast cancer in Ethiopia, and the only center to deliver comprehensive breast cancer care when data collection started. Breast cancer patients also present at private hospitals where a subset of breast cancer health care is delivered, so it is possible to miss some patients. The study design with retrospective data collection in Sweden and prospective data collection in Ethiopia could potentially introduce some biases, however, the retrospective design in Sweden was expected to be sufficient due to the rigid systems In Sweden regarding reporting and documentation in electronic patient charts. Despite of prospective data collection in Ethiopia, there are unfortunately missing data, especially regarding pathology results including NHG and IHC. This can partly be explained by the fact that breast surgery could be performed at other hospitals than TASH, making sample collection more difficult, and in some cases, patient took their specimen to private laboratories due to some waiting time for IHC in TASH. The fact that there was no ISH method available in TASH for verification of HER2 2 + cases is a limitation, and might have led to underestimation of the prevalence of the HER2-positive subtype. Although criterions for subtype classifications were aligned between study sites, there is a possibility that inter-laboratory variability could affect results, since there was no validation performed to ensure IHC consistency between pathology laboratories. Taking these methodology limitations into considerations, the difference seen in subtype distribution between groups should be interpreted with some caution and warrants further confirmatory studies.

## Conclusion

Young women and men with breast cancer present with similar symptoms, but in later stages of disease in Ethiopia, compared to in Sweden. The majority of breast cancers at both sites are ER-positive, and TNBC seem to represent only a small proportion in Ethiopia. Further studies and initiatives in LIC settings such as in Ethiopia need to focus on early detection to find cancer where cure is still an option. There is an urgent need to scale up the availability and the quality of pathology services in Ethiopia to make IHC available for all patients. Earlier diagnosis and timely treatment should allow for outcomes similar to those in Sweden, as tumor biology does not appear less favorable in the Ethiopian setting.

## Data Availability

The data that support the findings of this study are not openly available for reasons of sensitivity but are available from the corresponding author upon request. Data are located in controlled-access storage at the Department og Pathology, TASH, Addis Ababa, Ethiopia.

## Abbreviations

ASC-CAP: College of American Pathologists
ER: Estrogen receptor
HER2: Human epidermal growth factor receptor 2
H&E: Hematoxylin and eosin
HIC: High-income country
IHC: Immunohistochemistry
ISH: In situ hybridization
LMIC: Low-middle-income country
LIC: Low-income country
NHG: Nottingham grading
NKBC: Swedish National Quality Register of breast cancer
PgR: Progesterone receptor
SSA: Sub-Saharan Africa
SÖS: Södersjukhuset
TASH: Tikur Anbessa Specialized Hospital
TNBC: Triple negative breast cancer
TNM: Tumor node metastasis
WHO: World Health Organization
